# Uncomplicated *Plasmodium vivax* malaria in pregnancy associated with mortality from acute respiratory distress syndrome

**DOI:** 10.1186/1475-2875-13-191

**Published:** 2014-05-27

**Authors:** Rose McGready, Klanarong Wongsaen, Cindy S Chu, Nay Win Tun, Kesinee Chotivanich, Nicholas J White, François Nosten

**Affiliations:** 1 Shoklo Malaria Research Unit, Mahidol-Oxford Tropical Medicine Research Unit, Faculty of Tropical Medicine, Mahidol University, Mae Sot, Thailand; 2 Mahidol-Oxford Tropical Medicine Research Unit, Faculty of Tropical Medicine, Mahidol University, Bangkok, Thailand; 3 Centre for Tropical Medicine, Nuffield Department of Medicine, University of Oxford, Oxford, UK

**Keywords:** Acute respiratory distress syndrome, Maternal mortality, *Plasmodium vivax*, Severe malaria

## Abstract

The association between severe malaria and *Plasmodium vivax* species is contentious. On the Thai-Myanmar border, all pregnant women are followed systematically with active weekly malaria screening. Over a 27-year period of providing antenatal care, 48,983 have been prospectively followed until pregnancy outcome (miscarriage or delivery) and 4,298 women have had *P. vivax* detected at least once. Reported here is the first known *P. vivax*-associated death amongst these women. The initial patient presentation was of uncomplicated *P. vivax* (0.5% parasitaemia) in a term, multigravida woman who responded rapidly to oral artesunate and mefloquine treatment, clearing her blood stage parasites within 48 hours. The patient appeared well, was ambulatory and due to be discharged but became unwell with acute respiratory distress syndrome (ARDS) requiring ventilation three days (67 hours) into treatment. Despite induction and delivery of a stillborn foetus, ventilatory requirements increased and the patient died on day 7. The patient had a low body mass index. Sensitive detection with nested PCR confirmed only the presence of *P. vivax* species and concomitant infections such as tuberculosis and human immunodeficiency virus (HIV) were also ruled out. The contemporaneous treatment of acute uncomplicated *P. vivax* and the onset of ARDS on day 3 in this patient implies a possible but unconfirmed association with death in this patient. Assuming this death was caused by *P. vivax,* the risk of ARDS-related maternal mortality in this setting did not differ significantly between *Plasmodium falciparum* and *P. vivax* (0.24 per 1,000 (1/4,158) *versus* 0.23 per 1,000 (1/4,298), contrary to the increased risk of maternal mortality from *P. falciparum* compared to *P. vivax,* 2.89 per 1,000 (12/4,158) *versus* 0.23 per 1,000 (1/4,298), P = 0.003.

## Background

On the Thai-Myanmar border *Plasmodium falciparum*[1] and *Plasmodium vivax*[2] malaria are associated with adverse outcomes in pregnancy, with *P. falciparum* species typically involved in mortality reports [3]. Tests of lung function in both uncomplicated *P. falciparum* and *P. vivax* malaria have demonstrated a decrease in diffusion capacity which has been attributed to a reduction in the pulmonary capillary vascular component of gas transfer [4]. Following treatment, gas transfer is progressively reduced due to interstitial and alveolar oedema and is more prominent in *P. vivax* compared with *P. falciparum* infection. Sequestration of *P. vivax* in human lung tissue has not yet been shown, suggesting non-sequestering mechanisms for acute respiratory distress syndrome (ARDS), such as soluble mediators causing endothelial damage, hypoxia and systemic shock, resulting in diffuse alveolar-capillary damage [5,6]. Reported in this manuscript is a case of ARDS commencing on day 3 into treatment for uncomplicated *P. vivax* infection when the patient appeared to be in the recovery phase of the illness.

## Case presentation

In this case a 26-year old, gravida 3, parity 2, ethnic Mon pregnant woman from Myanmar first presented to the Shoklo Malaria Research Unit (SMRU) antenatal clinic (ANC) at 30 weeks and six days of pregnancy in early February 2013. This patient was a literate, non-smoker, with no history of prior medical problems whose prior obstetric history included one normal vaginal delivery and the most recent delivery was a caesarean section in Moulmein (Mawlamyine) for breech presentation. She was a migrant worker (migrating between Myanmar and Thailand) and resident for one month at her present address. At the first ANC visit, her vital signs and physical examination were normal except her body weight, which was low at 34 kg (BMI only 17.1 kg/m^2^ in late pregnancy). Her screening tests were negative for malaria, HIV, syphilis, and hepatitis B; and glucose-6-phosphate dehydrogenase status was normal. Her haematocrit (HCT) was low at 26% and haemoglobin typing confirmed she had β-thalassaemia trait, so ferrous and folic acid supplements were commenced. At routine weekly ANC consultations her malaria smear screening was negative a total of three times. Note in this area of the border with multidrug-resistant *P. falciparum* and *P. vivax*[7], women are actively and frequently screened by malaria smear (weekly and 24-hour service) and all positive cases are treated (regardless of symptoms) as there is no drug with proven safety and efficacy for prophylaxis during pregnancy.

At a gestation of 36 weeks and six days, six weeks (day 0) after booking, the patient complained of a seven-day history of fever, along with muscle, joint and abdominal pain, dizziness, palpitations, anorexia, and sleep disturbance. On examination the body temperature was slightly elevated at 37.7°C, blood pressure 100/65 mmHg, respiratory rate 26 breaths per minute (similar to six weeks earlier when it was 24 breaths per minute), pulse rate 90 per minute and the fetal heart rate was 134 beats per minute. Malaria smear showed a 0.5% (16,238/uL) *P. vivax* parasitaemia with all stages of the parasite present (Table 1). Her complete blood count showed a haemoglobin of 8.4 mg/dL, HCT 26.7%, mean cell volume 60.8 fl, white blood cells 1.5 × 10^3^/ml with differential counts (×10^3^/ml) of neutrophils (1.1), lymphocytes (0.3), monocytes (0.1), eosinophils (0) and basophils (0), and platelet count 138 × 10^3^/μL and C-reactive protein was 51.2 mg/L, consistent with a diagnosis of acute *P. vivax*. Microbiological examination of blood and urine, and scrub typhus IgG serology were negative. Treatment with fixed mefloquine and artesunate (Far-Manguinhos, Brazil) (artesunate/mefloquine 100 mg/220 mg) was started at one tablet once daily for three days with a good clinical response. This treatment was used in place of three days of chloroquine, the standard treatment, as the patient agreed to participate in a clinical trial [8]. Three supervised anti-malarial treatment doses were completed by 48 hours. The parasite count reduced rapidly (Table 1). The fever clearance time was 12 hours and the clinical symptoms reduced on day 1 with the patient only complaining of anorexia but less than on the previous day, and on day 2 anorexia (again less than the previous day) and new onset dizziness and palpitations.

**Table 1:** Parasite clearance with fixed mefloquine-artesunate once daily dosing

**Day**		**Parasite count**		**Parasitaemia/uL**
**(hour)**	**Trophozoites**	**Schizonts**	**Gametocytes**	
0 (0)	*P. vivax* 5/1,000 RBC	*P. vivax* 55/500 WBC	*P. vivax* 10/500 WBC	16,238
1 (21)	*P. vivax* 4/500 WBC	*P. vivax* 1/500 WBC	*P. vivax* 75/500 WBC	12
2 (48)	Negative	Negative	*P. vivax* 3/500 WBC	n a
3 (65)	Negative	Negative	*P. vivax* 1/500 WBC	n a

na: not applicable as calculated for trophozoite only.

On day 2, the patient was well and she was due to be discharged the following day. Around midnight on day 2 she complained of a small amount of haemoptysis and on day 3 at 65 hours there was a significant deterioration in the clinical condition of the patient. Her vital signs included a temperature of 36°C, blood pressure 130/80, respiratory rate of 39 breaths per minute, heart rate of 90 and oxygen saturation of 40% on room air. Dyspnoea and haemoptysis were not associated with chest pain and symptoms were somewhat relieved by the upright position, not recumbent. This was improved to 92% with facial mask oxygen at a flow rate of 10 L/minute. Malaria smear was negative with only *P. vivax* gametocytes detected (Table 1). A complete blood count showed a haemoglobin of 7.2 mg/dL, HCT 22.6%, white blood cell count 5.5 × 10^3^/ml with a differential counts (×10^3^/ml) of: neutrophils (4.4), lymphocytes (0.8), monocytes (0.3), eosinophils (0) and basophils (0), and platelet count of 178 × 10^3^/μL. The C-reactive protein was 80.8 mg/L, sodium 131.7 mEq/L, potassium 3.58 mEq/L, urea nitrogen 10.2 units, creatinine 1.0 mg/dL, total bilirubin 1.58 mg/dL, direct bilirubin 0.74 mg/dL, AST 67 U/L, ALT 22 U/L, alkaline phosphatase 588 u/L, total protein 6.5 g/dL, and anion gap 15.2 mmol/L, all of which were considered to be consistent with recovering acute malaria.

Intensive care was not available at the field hospital where the patient had been admitted and the patient was referred to the district hospital (75 minutes by road) where she was intubated in the casualty department and admitted to the intensive care unit. Arterial blood gases obtained after intubation with 100% oxygen revealed a pH of 7.32, PaCo2 34 mmHg, a base excess -8.7 mmol/L, and SaO2 87%. Electrocardiogram was normal as was an echocardiogram which showed normal right-sided heart pressures and volumes, and a chest radiograph showed bilateral pulmonary (fluffy) infiltrates. Two separate sputum tests for acid fast bacilli were negative. Treatment with artesunate, clindamycin, chloramphenicol, ceftriaxone, and frusemide was commenced. An induction on the second day of hospitalization (day 4 since first diagnosis of *P. vivax*) for known fetal death *in utero* resulted in a forceps delivery of a stillborn 2,500 g normal male infant. Unfortunately, after delivery ventilator pressures continued to increase [9]. On day 5 her condition worsened with bilateral pneumothorax. A CT scan with contrast done after insertion of bilateral chest drains reported ground glass opacity of the lung tissue and no pleural effusion. The patient had a cardiac arrest later the same day and died on day 7. No autopsy was done.

### Species PCR

Nested PCR was performed on DNA extracted from 565 uL of packed red blood cells obtained just prior to treatment, following the protocol [10] with the following exceptions: the QIAamp DNA blood mini kit (Qiagen, Germany) was used to extract the DNA and Bioline reagents (BIOTAQ™ DNA polymerase) were used to set up 20 μl PCR reactions. The sensitivity of the nested PCR assay at SMRU is 1 parasite/uL of whole blood for *P. falciparum*; 1 parasite/uL of whole blood for *P. vivax*; 10 parasites/uL of whole blood for *Plasmodium malariae* and 6.3 parasites/uL of whole blood for *Plasmodium ovale*. Using these methods the sample was found to be positive only for *P. vivax* and negative for other *Plasmodium spp.*

### Acute respiratory distress syndrome, malaria and pregnancy

While sequestration of *P. vivax* in the lung circulation has been suggested clinically [4] but not proven [6,11] there are *in vitro* conditions where adherence of *P. vivax*-infected red blood cells (RBC) has been demonstrated (immobilized chondroitin sulphate A and hyaluronic acid and fresh placental cells [12]; chondroitin sulphate A and intracellular adhesion molecule-1 receptors on human lung endothelial cells, Saimiri brain endothelial cells and placental cryosections [13]) although to a lesser extent than observed with *P. falciparum*. In non-pregnant adults, *P. vivax*-associated ARDS typically occurs after parasitaemia has declined on day 2 or 3 and a review of published case reports indicate that rapid intubation and ventilation are important for survival and cases that have survived were not pregnant (91.7% (22/24)) [5,14]. In a broad review of the clinical spectrum or *P. vivax*, ARDS as a single complication has been reported in low numbers over the last 20 years, mainly from high income, non-endemic countries with a low case fatality rate. This is contrary to much higher rates (case fatality rate of 50-67%) from more recent reports from endemic countries of severe vivax with ARDS occurring as part of multiple organ dysfunction/failure [15]. The risk of ARDS in relation to uncomplicated *P. vivax* has not been quantified, while for *P. falciparum* it has been estimated to be 0.1% (three in 3,300) US Army soldiers in Vietnam [16].

Systematic malaria screening (and treatment when positive) of all pregnant women at weekly antenatal consultations was introduced in 1986 at SMRU to reduce *P. falciparum* malaria-related maternal mortality [3] which was estimated at 1% of all pregnant women annually [2]. Since then and until the patient in this case died, there were 48,983 prospectively followed pregnancies with a known outcome (birth or miscarriage) and 12 *P. falciparum* and one *P .vivax* (the case in point) related maternal deaths, with each species accounting for one ARDS death. Amongst this cohort of pregnant women, malaria screening detected at least one episode of *P. falciparum* or *P. vivax* in 4,158 and 4,298 women, respectively. Assuming the case in point was caused by *P. vivax* the comparative rates of malaria-related mortality were: *P. falciparum* 2.89 per 1,000 (12/4,158) and *P. vivax* 0.23 per 1,000 (1/4,298), P = 0.003; whereas the ARDS-related malaria mortality was not significantly different for *P. falciparum* 0.24 per 1,000 (1/4,158) and for *P. vivax* and 0.23 per 1,000 (1/4,298) P = 1.000.

Reviews of cases suggest *P. vivax*-associated ARDS is mostly reported to occur after the start of anti-malarial treatment and potentially more cases will be seen with the spread of chloroquine-resistance and use of alternative regimens without the same anti-inflammatory drug properties [15]. However, mefloquine, which was the partner to artesunate in this case, also has anti-inflammatory properties [17].

## Conclusion

The maternal death reported here is highly unusual in the context of the very high numbers of prospectively followed pregnancies with a confirmed diagnosis and treatment of *P. vivax* in this population. The patient was in late pregnancy, symptomatic, moderately anaemic with known β-thalassaemia trait and with a relatively high *P. vivax* parasitaemia. She was treated for uncomplicated malaria infection, took oral artemisinin-based anti-malarials and appeared to be recovering well. Unexpectedly ARDS developed on day 3 after treatment was completed and when trophozoites were no longer detectable on the peripheral blood smear. Although ventilated, she died from complications of ventilation. While no other cause for ARDS was diagnosed in this patient, she did have a low body weight and without autopsy an underlying condition cannot be completely ruled out. While most *P. vivax*-associated ARDS cases reported in the literature have recovered, they were not pregnant. This case of ARDS-associated mortality may have been related to the original *P. vivax* infection but this cannot be proven. If it is assumed that the patient’s cause of death was caused by *P. vivax*-associated ARDS, then the risk of ARDS-related maternal mortality in this setting was similar for *P. falciparum* and *P. vivax* infection, contrary to malaria-related mortality which was more than 12-fold higher with *P. falciparum*.

## Consent

Written informed consent was obtained from the patient for publication before she died as she was enrolled to a study ( http://clinicaltrials.gov/ct2/show/NCT01054248) approved by the Oxford Tropical Research Ethics Committee (reference 45–09) and the Faculty of Tropical Medicine, Mahidol University, Thailand (reference MUTM 2009-065-01). A copy of the written consent is available for review by the Editor-in-Chief of this journal.

## Abbreviations

ANC: Antenatal clinic; ARDS: Acute respiratory distress syndrome; HCT: Haematocrit; HIV: Human immunodeficiency virus; SMRU: Shoklo malaria research unit.

## Competing interests

The authors declare they have no financial competing interests to declare. One of the authors (RM) is on the editorial board of *Malaria Journal*.

## Authors’ contributions

CC and NWT managed the patient. KW contributed to the laboratory work. RM, NJW and FN conceived of the particular study to which the patient was enrolled. RM, KW, CC and NWT prepared the first draft of the manuscript. All authors were involved in the revision of the draft manuscript and have read and approved the final manuscript.
